# A nonrandomised feasibility evaluation of a home‑based intervention for parents of adolescents with learning disabilities, with or without autism

**DOI:** 10.1007/s00431-026-07246-1

**Published:** 2026-07-17

**Authors:** Michael Absoud, Carla Rush, Lisa Mackey, Amy Waters, Gordon Forbes, Karen Pratt, Ashley Liew, Katy Strudwick, Vicky Slonims

**Affiliations:** 1 Evelina London Children’s Hospital, King’s College London, London, England; 2 Department of Women & Children’s Health, King’s College London, Children’s Neurosciences, Becket House, London, SE1 7EH England

**Keywords:** Learning disabilities, Autism, Adolescents, Parent‑mediated intervention, Home‑based therapy, Feasibility study

## Abstract

This study aims to determine the feasibility and acceptability of delivering ACHIEVE—a home‑based, parent‑coached OT/SLT intervention—in everyday UK practice and to describe recruitment/retention, data completeness, working alliance, delivery logistics, and exploratory changes in parent‑reported functional, behavioural, communication and stress outcomes. Single‑arm, non‑randomised feasibility study aligned with CONSORT pilot/feasibility guidance. Thirty‑six families of adolescents (11–19 years) with moderate–severe learning disabilities (many also autistic) were recruited via three special schools. Individually tailored OT/SLT sessions focussed on parent‑nominated goals in self‑care/daily living, communication, and behaviour. Hybrid delivery (home visits + secure video); feasibility and acceptability were primary outcomes; exploratory secondary outcomes included DBC, HSQ, APSI, COPM, and GAS. Recruitment via schools was via a convenience sample (36 families). Attendance was high (159/190 sessions; 83.7%) with 99 home and 59 video sessions. Endpoint data were obtained for 22 families. Parents reported strong alliance (WAI median 89.5, IQR 73.0–91.0). Exploratory signals suggested improvement: DBC *T*‑score − 3.90 (*p* = 0.004; *n* = 19); HSQ mean severity − 0.71 (*p* = 0.018; *n* = 19); APSI total − 6.80 (*p* = 0.046; *n* = 20). COPM median change + 3.0 (performance) and + 4.0 (satisfaction) (both *p* < 0.001; *n* = 17). GAS modelling suggested dose effects: ~ 7 sessions linked to ~ 80% probability of achieving expected goals, and ~ 10 to exceeding them (*n* = 31).

*Conclusion*: ACHIEVE was feasible and acceptable in UK practice with high engagement and manageable data collection. Dose–response signals and alliance strength support a minimum session count and continued coaching emphasis. Findings inform outcome selection, dose, and schedules for a definitive trial; clinical changes remain exploratory.

**Table:** 

**What is Known:** • *Adolescents with learning disabilities (often with co-occurring autism) and their families struggle to access practical home‑based support; robust evidence is limited*. • *Family‑centred, parent‑coached allied‑health interventions are promising but under‑studied for adolescents and in home/hybrid formats*.	
**What is New:** • *ACHIEVE—a home‑based Occupational therapy/Speech & Language Therapy, parent‑nominated goal programme—proved feasible and acceptable with strong therapeutic alliance for families of adolescents with learning disabilities with or without autism*. • *Exploratory improvements and a dose signal (~7–10 sessions) guide scheduling and outcomes for a future comparative trial*.

**What is Known:**

• *Adolescents with learning disabilities (often with co-occurring autism) and their families struggle to access practical home‑based support; robust evidence is limited*.

• *Family‑centred, parent‑coached allied‑health interventions are promising but under‑studied for adolescents and in home/hybrid formats*.

**What is New:**

• *ACHIEVE—a home‑based Occupational therapy/Speech & Language Therapy, parent‑nominated goal programme—proved feasible and acceptable with strong therapeutic alliance for families of adolescents with learning disabilities with or without autism*.

• *Exploratory improvements and a dose signal (~7–10 sessions) guide scheduling and outcomes for a future comparative trial*.

## Background

In the UK, around 353,000 children (approximately 2.5%) have a learning disability. About half of these children are also autistic, and roughly one quarter of autistic children and young people have co‑occurring learning disabilities. Learning disabilities increase the risk of ill-health and are associated with a shorter life expectancy than in those without a learning disability [1]. In addition, young people with learning disabilities are more likely to engage in behaviours that challenge others and are 3–4 times more at risk for mental health problems [2–5]. These challenges can negatively affect parent–child relationships [5–8] and families in these circumstances often struggle to obtain the help they need [6]. In some cases, escalating challenges may necessitate young people with learning disabilities leaving the family home to live in residential provision [7].

Difficulties with the development of adaptive skills are a core component of the diagnosis of learning disabilities, but there is little research exploring the possible benefits of interventions to improve adaptive skills in this population [8]. Most studies involved very few participants, often caregivers in the home context, and authors recommended more research on the process of goal setting [9]. A scoping review of occupational therapy interventions to improve daily living skills in autistic adolescents identified common themes such as generalisation of skills to different contexts, maintenance of skills, incorporating family perspectives, and the use of technology [10]. Although communication impairment is common in people with learning disabilities [11], there is little evidence exploring the efficacy of speech and language therapy with this group. Speech and language therapists working with people with profound or multiple learning disabilities describe their interventions as being focussed on the empowerment and development of an individual, taking into account their behavioural preferences, but research is rarely influential in the selection of interventions, and there is very diverse practice between professionals [12]. A review of speech and language therapy interventions with adults with learning disabilities rated most studies as low quality with small numbers of participants and often no comparison groups [13]. The authors reported difficulty in identifying interventions which focus exclusively on communication targets.

Hierarchy of family involvement models have examined how professionals engage and collaborate with families and have identified a continuum of involvement [14]. At the lowest level, parents can be seen as deficient recipients of expert care, to the highest level where families make informed choices about the focus and type of support they receive, with professionals acting as agents of the family. However, many therapy services involve parents as the implementers of activities and would need to alter their approach to achieve co-working in intervention [15]. Family-centred models providing a collaborative approach to working with parents take account of contextual factors, e.g. accommodating parents’ commitments, mechanisms such as relational aspects, the role of active help-giving, the value of co-designed goal and support such as coaching focussing on parent nominated outcomes [16]. Parents’ belief in the intervention and trust in the therapist is increased when interventions are collaborative, and relational factors are considered [17]. Key ingredients include finding shared terminology and ‘naming’ skills and problems and working on activities together. These factors increase a parent’s sense of control and empowerment, as well as their knowledge, skills, and capability beliefs [18].

Home-based and parent-mediated interventions and approaches aim to build caregiver confidence, knowledge, and capability so that strategies can be embedded into everyday routines, increasing opportunities for practice, generalisation, and maintenance in natural contexts. This is particularly relevant for adolescents with learning disabilities and/or autism, whose challenges with communication, self-care, participation, and behaviours that challenge often occur in the home and community rather than in clinic settings. Evidence from parent-mediated interventions in autism suggests potential benefits for behaviours that challenge and adaptive functioning, although the certainty of evidence remains limited and further trials are needed [19, 20]. Existing evidence for parent interventions in adolescents with learning disabilities suggests potential benefits for parenting, parent–adolescent relationships, and wellbeing, but the evidence base remains heterogeneous and there is little cost-effectiveness evidence [21]. The ACHIEVE study (AdolesCents Home based InterVEntion Study) was therefore developed as a pragmatic, family-centred and home-based intervention to address an important gap in support for adolescents with moderate to severe learning disabilities, many of whom are also autistic.

The ACHIEVE study hence aimed to:

- Enhance parents’ confidence in their ability to support their child
- Assess the feasibility of delivering a home-based intervention
- Work with parents on their nominated targets:
  o Functional skills
  o Communication skills
  o Reduction of behavioural difficulties
- To understand family members’ perspectives on this type of care using qualitative measures

## Methods

### Study design

The ACHIEVE study was a single group feasibility study assessing the impact of a home-based intervention in children and young people with moderate or severe learning disabilities.

The ACHIEVE project was developed by an NHS clinical service in response to parent feedback that families of adolescents who have learning disabilities (LD) and/or autism were not receiving direct help at home. This project is a family-informed innovative therapy pathway comprising NHS provision in the family home.

Following guidance on the conduct of pilot and feasibility studies [22], we designed the study to assess the feasibility of recruitment and retention to the intervention and the feasibility of data collection in this population.

### Participants

We intended to approach 60 adolescents recruited from 3 special schools for pupils with learning disability (they may also be autistic) in the South East of England (November 2021–June 2023). Schools were asked to notify families where they were aware that parents were experiencing difficulties with their child at home. We also asked school staff to consider if parents had adequate English to understand and consent to the requirements of the study. Easy-to-read documents were designed to facilitate this.

### Inclusion criteria

- Children and young people aged between 11 and 17 years
- Children and young people with severe–moderate learning disabilities and may also be autistic, as stated on their Education, Health and Care Plan
- Children and young people attending a special educational needs school
- Children and young people whose parents/carers consent to participation

### Exclusion criteria

- Children and young people with profound and multiple learning disabilities
- Children and young people with current child protection issues where the intervention could interfere with social service interventions and/or there could be an unsafe environment for the therapists

### Ethical approval

Approval was obtained from London–Queen Square Research Ethics Committee on 19 October 2021, REC reference: 21/LO/0645, IRAS project ID: 299,406. The study was registered with the ISRCTN (ACHIEVE: www.isrctn.com/ISRCTN28527653).

### Procedure

Special schools familiar to the clinical service in Greater London agreed to assist with recruitment. Recruitment via the NHS was not considered feasible as many adolescents with learning disabilities do not receive ongoing health care unless they have a mental disorder or physical illness. Senior managers in schools signed a memorandum of agreement prior to the distribution of recruitment packs, including consent forms to parents/carers of pupils meeting inclusion criteria. Schools then distributed recruitment packs to eligible parents of pupils meeting inclusion criteria via the home-school bag. For ethical and practical reasons, parents returned their responses in sealed envelopes to the school office so that school staff were not aware of their decision about participation in the study. Envelopes were collected by members of the research team who then contacted the parents to explain the study in detail and obtain consent.

The intervention is described according to TIDieR protocol [23]:

- Study outcomes: ACHIEVE (AdolesCents Home based InterVEntion Study)—improving self-care for adolescents with learning disabilities
- Why: To provide a home-based, individually tailored solution focussed intervention focussing on adaptive or communication skills and any related behavioural challenges.

ACHIEVE was compiled by an NHS specialist clinical academic team in response to parent-identified gaps in practical support for adolescents with learning disabilities and/or autism at home. The intervention drew on family-centred care, parent coaching, collaborative goal-setting, and allied-health approaches to adaptive functioning, communication, and participation. Occupational therapy and speech and language therapy were selected because the anticipated goals related primarily to self-care and daily living skills, communication, environmental/sensory adaptation, participation in home routines, and behaviours that challenge. ACHIEVE therefore uses manualised core components (including joint assessment, collaborative goal-setting, and caregiver coaching) while allowing tailoring to each family’s priorities and context.

- What:
  - Materials: Parents were provided with materials tailored specifically to their focus for intervention e.g. written guidance on managing behaviour, laminated pictures or symbols, images of visual timetables or printed information sheets e.g. sleep hygiene information. Parents were also given charts to keep records of their child’s progress (Goal Attainment Scales records)
  - Procedures: Therapists and parents engage in a supportive conversation in which the family describe their experiences of family life with their adolescent. ACHIEVE includes manualised core components (joint assessment in the home, collaborative goal setting, and caregiver coaching) delivered flexibly and tailored to each family’s priorities, within a structured OT/SLT framework. This provides the context for a formulation-driven clinical approach in which the therapist uses their expert knowledge, skills and experience to establish family driven practical approaches to managing situations at home.

- Who provided: The therapy was delivered by a band 7 (highly qualified) specialist occupational (OT) or speech and language therapist (SLT) with expertise and experience in working with young people with learning disabilities and autism. Prior to delivery they received training in the ACHIEVE framework, including joint home assessment, collaborative goal-setting, caregiver coaching, use of COPM and goal attainment scaling, structured therapy records, documentation of tailoring, safeguarding/lone-working procedures, and secure video delivery. Weekly supervision with senior clinicians reviewed goals, therapy content, tailoring, parent engagement, and progress against the ACHIEVE framework.
- How delivered: The first session was always delivered by the two therapists in the family home to meet the family before their child returned from school. The meeting focussed on hearing the parent’s perspectives for shared understanding of perspectives. The therapists were present when the adolescent returned home from school, giving them the opportunity to meet the individual and to understand the context. For some families, it was possible to establish goals for intervention in this first session, but it often required at least one more session to do this fully. Both therapists (OT and SLT) were able to deliver targets focussed on adaptive and communication skills and supervision and shared case discussion allowed for trans-disciplinary working; however, a single therapist was allocated to work with the family based on the primary focus for intervention.
- Where: After the initial home visit, sessions with the family were via video link or home visit according to family preference. The adolescent was sometimes kept at home so the family could demonstrate how they are using the new techniques or to problem-solve issues.
- When and how much: Initial sessions were 60–90 min in duration, and follow-up sessions were about 30–60 min. Travel time was included in the workflow formulations for therapy staff. At least 4 out of 10 possible sessions were conducted in the home, and the final session was always a home visit.
- Tailoring and modifications: All interventions were individually tailored to the family, with a maximum of 3 goals. Goals varied in complexity and the time needed to achieve them. New strategies were developed in response to individual family styles or specific child factors, e.g. the involvement of a sibling in therapy, and these were recorded in the therapy log.
- How well: Therapists used a structured session record to document (i) goals addressed, (ii) strategies introduced or rehearsed, and (iii) tailoring/modifications. Fidelity to core components and consistency of delivery were supported through weekly supervision with senior clinicians, where goals, session content, and progress were reviewed against the intervention framework. After each session, therapists recorded a brief rating of parent engagement and use of strategies discussed (as an indicator of adherence in the home context). Independent observational fidelity coding (e.g. third-party coding of recorded sessions) was not undertaken in this feasibility evaluation and is planned for future controlled studies.

### Measures—primary outcomes

The primary goal of the study was to establish the feasibility and acceptability of delivering the ACHIEVE intervention at home. Evaluation focussed on the recruitment and retention of participants, data collection rates, information on the delivery of the therapy at home (including Research OT and SLT rated parent fidelity to treatment), process evaluation, and qualitative data on the experiences of families and the research therapists. Secondary exploratory outcomes focussed on potential treatment effects. An exploratory examination of the association between treatment dosage and changes in targeted behaviours was also conducted.

1. Feasibility of the intervention

Detailed information was collected on:

a Treatment delivery – i.e. numbers of treatment sessions offered, delivered and cancelled, and fidelity of therapy delivery by parents.
b Completion rates for assessment questionnaires (from parents) at baseline to end point of the intervention.

2. Family and child characteristics

Demographic data (baseline only): The parents were interviewed and asked about child and family ethnicity, maternal education, family social-economic level (derived from the Index of Multiple Deprivation based on postcodes) (Ministry of Housing 2019), and the number of children in the family*.*

3. Family use of other provision/interventions

The Child and Adolescent Service Use Schedule (CA-SUS) was completed by parents at the end of the treatment period to provide information about local service use by families (e.g. speech and language therapy, alternative medicines, and other education provision) [24].

4. Acceptability of therapy: parents’ experiences/engagement in therapy

Included therapeutic alliance, parenting sense of self-efficacy and barriers to treatment.

### Measures—secondary outcomes

Pre-post evaluation of the therapy on parent-rated measures administered by Research Assistant:

- Home Situations Questionnaire (HSQ) domain: Parental questionnaire designed to assess behavioural non-compliance in everyday settings [25]. It gathers specific information from parents about behaviours and symptoms (Cronbach’s α = 0.84).
- The Developmental Behaviour Checklist (DBC)[26]:
  Domain: Parental questionnaire on child’s behaviour (Cronbach’s α=0.94).
- Autism Parenting Stress Index (APSI) to assess stress in parents of children with Autism Spectrum Disorder (ASD) across domains to assist in identifying support needs and track intervention effectiveness (Cronbach’s α = 0.83)) [27]

Pre-post evaluation of the therapy focussed on parent nominated targets for intervention, i.e. adaptive, behavioural, and communication skills. These were integral to the therapy, hence administered by the research OT and SLT.

- Goal Attainment Scales (GAS) [28]
- Canadian Occupation Performance Measure (COPM) [29]

The Goal Attainment Scale allowed each participant to set up to three goals.

**Fig. 1:**
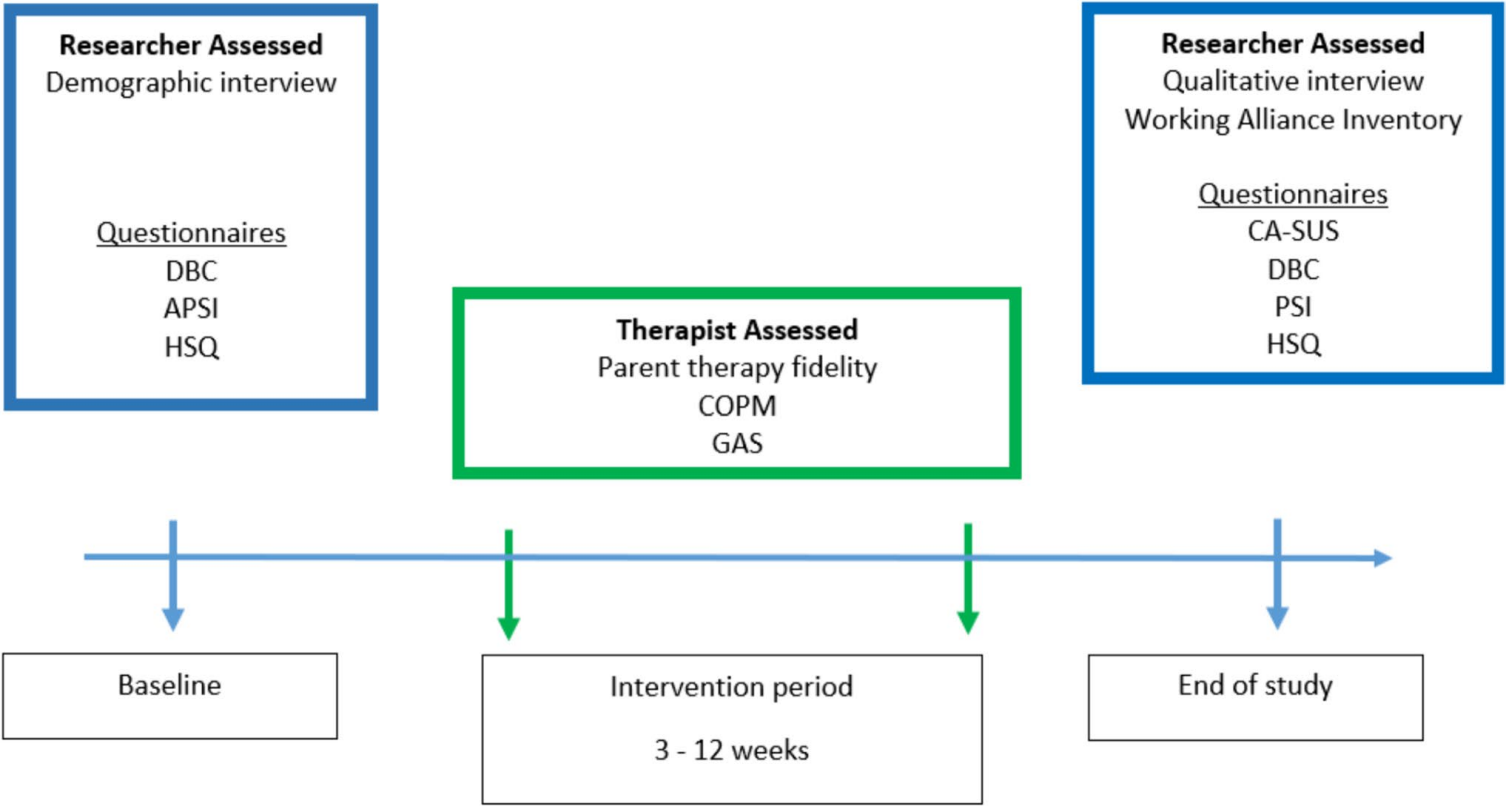
Timing of assessments for an individual participant

*Timing of assessments*: Fig. 1 illustrates the time points at which the different assessments were completed.

### Statistical analysis

Feasibility outcomes are reported using descriptive statistics. Baseline and post-intervention scores were compared using a paired *T*-test for DBC, HSQ, and ASPI. Changes in the COPM were analysed using a Wilcoxon signed rank test. Participants were included in the analysis if they had complete data at baseline and outcome. The association between GAS assessed at the end of each therapy session and the number of sessions of therapy delivered was analysed using a proportional odds model, with sessions number as predictor, and cluster robust standard errors used to account for clustering by individual and goal.

## Results

### Feasibility and acceptability of ACHIVE intervention at home

**Table 1:** Study sample demographics

Variable	*n* (%)
Child characteristics (***N*** = 34)	
Sex—female	12 (35%)
Sex—male	22 (65%)
Ethnicity—White	8 (24%)
Ethnicity—mixed/multiple backgrounds	4 (12%)
Ethnicity—Asian/Asian British	5 (15%)
Ethnicity—Black/African/Caribbean/Black British	11 (32%)
Ethnicity—not reported at baseline	6 (18%)
Nominated parent characteristics (***N*** = 34)	
Gender—female	32 (94%)
Gender—male	2 (6%)
Ethnicity—White	10 (29%)
Ethnicity—mixed/multiple backgrounds	1 (3%)
Ethnicity—Asian/Asian British	5 (15%)
Ethnicity—Black/African/Caribbean/Black British	12 (35%)
Ethnicity—not reported at baseline	6 (18%)
Nominated parent highest qualification (***N*** = 34)	
Degree or equivalent	9 (26%)
Higher education	6 (18%)
A level	5 (15%)
GCSE	5 (15%)
Other qualification	1 (3%)
No qualification	8 (24%)
Other parent highest qualification (if reported) (***N*** = 34)	
Degree or equivalent	16 (47%)
Higher education	4 (12%)
GCSE	5 (15%)
No qualification	9 (26%)
Household composition	
Number of children—1	7 (21%)
Number of children—2	14 (41%)
Number of children—3 +	7 (21%)
Not reported at baseline	6 (18%)
Languages at home	
English main home language	23 (of 28 respondents; 82%)
Other language(s) spoken	17 (of 28 respondents; 61%)
Indices of multiple deprivation (IMD) deciles (***N*** = 34) §	
Decile 1 (most deprived)	0 (0%)
Decile 2	7 (21%)
Decile 3	17 (50%)
Decile 4	4 (12%)
Decile 5	1 (3%)
Decile 6	1 (3%)
Decile 7	0 (0%)
Decile 8	2 (6%)
Decile 9	1 (3%)
Decile 10 (least deprived)	1 (3%)

#### Recruitment of participants

A convenience sample of 36 children (12 female) was recruited across 3 schools (Table 1). Median age was 15 years old (range 11–19). There were 2 withdrawals and data available for 34 children.

### Retention of participants

**Fig. 2:**
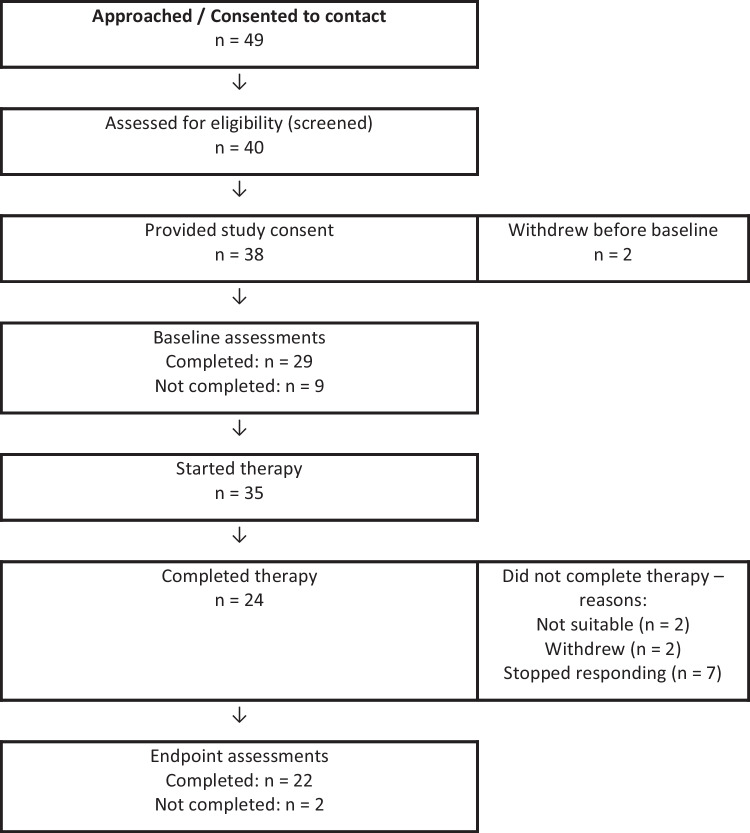
Consort diagram—family recruitment

Twenty-four families completed therapy (Fig. 2). Initially, families completed consent and baseline/endpoint questionnaires via post, but this was later modified so that all families were visited at home by the researcher for consent and baseline to help with queries. Subsequently, home visits or Teams appointments were used for endpoint evaluations.

1. Completion rates for assessment data (Table 2)

**Table 2:** Questionnaire completion

Questionnaire	Baseline (*n*)	Endpoint (*n*)
APSI	28	22
HSQ	26	21
DBC	26	22
COPM	17	17
GAS	—	25
WAI	—	22

*APSI*, Autism Parenting Stress Index; *HSQ*, Home Situations Questionnaire; *DBC*, Developmental Behaviour Checklist; *COPM*, Canadian Occupation Performance Measure; *GAS*, Goal Attainment Scales; *WAI*, Working Alliance Inventory
2. Delivering the intervention at home
  A total of 190 therapy sessions were booked, of which 159 were attended (an attendance rate of 83.7%), comprising 99 face-to-face sessions, 59 video sessions, and 1 phone session. Communication activity with families included 158 contact calls, 396 text messages, and 174 emails.
  The feasibility checklist was not formally adapted by demographic characteristics or autism status. Tailoring was instead documented through parent-nominated goals and therapy records. Goals clustered around: daily living/self-care independence, including dressing, bathing, toileting, toothbrushing, and eating/drinking skills; routines and schedules, including morning/after-school routines, sleep, and visual timetables; communication and choice-making, including picture/photo boards, PECS, talking mats, and objects of reference; community participation, including shopping, park visits, and transition to college; and behavioural regulation/flexibility, including tolerating unexpected change and reducing outbursts around self-care tasks. Adaptations commonly included visual supports, task breakdown, graded practice, environmental modification, structured routines, and parent coaching. These were selected by individual formulation rather than diagnosis alone, although visual, routine-based, and transition-focused strategies were particularly relevant for adolescents with co-occurring autism.
3. Change data from key measures (Table 3)

**Table 3:** Baseline vs. endpoint summary with paired *T*-tests for DBC, HSQ, and APSI

Measure	Baseline mean	Baseline SD	Endpoint mean	Endpoint SD	Mean difference	*T*-statistic	*p*-value
DBC total *T*-score	61.9 (*n* = 26)	10.44	59.1	11.42	− 3.90 (*n* = 19)	3.26	0.004
HSQ mean severity	5.24 (*n* = 26)	1.67	4.51	2.06	− 0.71 (*n* = 19)	2.60	0.018
APSI total	32.1 (*n* = 28)	14.10	25.1	12.93	− 6.80 (*n* = 20)	2.14	0.046

Developmental Behaviour Checklist (DBC); Home Situations Questionnaire (HSQ); Autism Parenting Stress Index (APSI)

#### Canadian occupational performance measure (COPM)

COPM performance (*n* = 17) median change was + 3.0 (95% CI = 2.0–4.0; range 0–8); Wilcoxon Signed-rank test *p*-value < 0.001. COPM satisfaction (*n* = 17) median change was + 4.0 (95% CI = interval: 3.0–4.0; range 0–9); Wilcoxon signed-rank test *p*-value < 0.001.

#### Goal attainment scales (GAS)

**Fig. 3:**
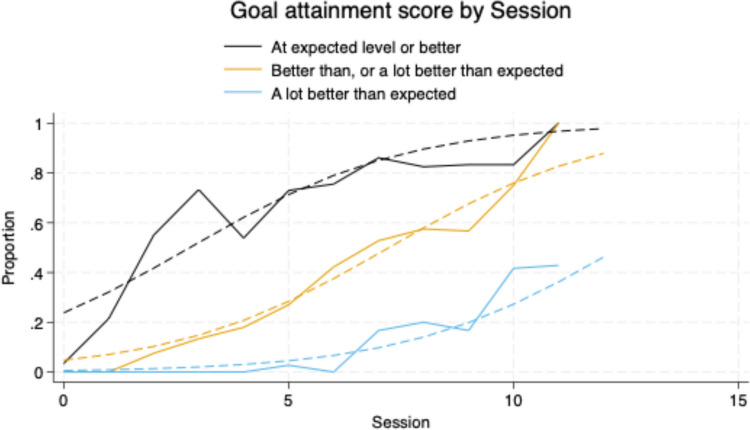
Cumulative probability of meeting or exceeding goals vs. number of sessions delivered. Solid lines show observed proportions of participants at each level. Dashed lines show predicted probabilities from a proportional odds model

The cumulative probability of attaining different scores on the GAS score (range − 2 to + 2), by number of therapy sessions (Fig. 3). Seven sessions were required for a cumulative probability of 80% in achieving the expecting level of outcome improvement, and 10 sessions for a cumulative probability 80% for achieving a better or a lot better than expected outcome (*n* = 31).

#### Working alliance inventory (WAI)

The Working Alliance Inventory (WAI) total scores (*n* = 22) had a median of 89.5 with an interquartile range (IQR) of 73.0–91.0. Scores cluster high, indicating generally strong therapeutic alliances overall.

## Discussion

This feasibility trial demonstrates that a home-based, parent-coached programme for adolescents with learning disabilities can be delivered in everyday UK practice, that families engage with it, and that a hybrid home telehealth format is workable. We reached families through special schools, most started therapy, almost four in five booked sessions were attended, and parents reported a strong working alliance with therapists. Endpoint data were obtained for the majority of participants treated, and measures of changes on self-rated parenting stress and satisfaction, parent-rated behaviour, including their child’s level of compliance, and functional performance moved in the expected direction. Goal-attainment modelling suggested that dose matters. Around seven sessions were linked to meeting expected goals and closer to ten to exceeding them. This study provides feasibility work focussing on uncertainties, context, and helping to develop a programme theory that would support larger scale evaluation of effectiveness. As such, it accords with guidance to prioritise feasibility before effectiveness and to design evaluations that explicitly consider setting, logistics, and burden [22, 30].

With regard to implementation considerations, ACHIEVE required a flexible hybrid model, with substantial contact beyond scheduled sessions (calls, texts, and emails) alongside home and video appointments. For scale-up, key practical considerations include workforce (experienced OT/SLT able to work trans diagnostically), protected supervision time, travel/lone-working arrangements for domiciliary visits, and maintaining flexibility around family availability. A hybrid delivery option may improve access while reducing travel burden, but services will need systems for scheduling, safeguarding escalation routes, and materials provision to support family-tailored strategies.

This study has recognised that the context in which families of young people with learning disabilities live is important and cumulative exposure to social disadvantage further increases the risk of psychiatric disorder and limits participation in routine services [31]. This makes parent-facing support at home both necessary and pragmatic. Our findings suggest that it is feasible to deliver allied health professional expertise to families and coach parents on goals they nominate that fit everyday life without requiring time-consuming clinic attendance [21].

The ACHIEVE study directly addresses the focus of Integrated Care Systems ensuring joined up care [32]. It was delivered by allied health clinicians within NHS pathways with pupils recruited directly from diverse special schools. Its targets (self-care, communication, and behaviour in everyday routines) align closely with children’s health and social care priorities for promoting participation and independence and preventing escalation to high-cost provision. Delivery in family homes, coaching parents on their nominated goals, and hybrid scheduling to fit caregiving demands reflect the needs of families (family-centred, context-first, capacity-building). Importantly, recruitment via special schools, key multi-agency nodes, demonstrated that families can be identified and supported without additional clinic attendance, a practical fit for family centred, health, and social care commissioned support that occurs around school and home.

Reviews of programmes for parents of adolescents with learning disability report positive effects on parenting and parent-adolescent relationships, while simultaneously calling for stronger designs and better reporting [33]. Our specification of ACHIEVE using TIDieR and our fidelity plan respond directly to those gaps, increasing replicability and paving the way for scale-up.

ACHIEVE adds three elements that are often missing: first, the focus on adolescents, a group under-represented in parent-mediated trials compared with younger children, despite more complex school and social demands in readiness for adulthood; second, it works with families in their homes and local communities, through parent-nominated goals, which improves ownership and acceptability; third, it answers feasibility questions that are often unclear: how many sessions are needed, how families prefer to meet, how often staff can travel, and how to assist families without burdening households.

Interpretation of our clinical signals remains cautious, as it should in a feasibility phase. Parent-rated indices improved across the sample, and the dose–response data from goal attainment will help in future session scheduling. However, the single-arm design of the study does not allow us to establish the effects of the intervention. Our feasibility data hence tentatively allows power analysis for sample size calculations and our exploratory findings will help refine therapy delivery (i.e. number of sessions), select, and sequence assessments so that the next trial is more likely to successfully complete and report.

Our feasibility records also informed a process evaluation describing modifications made during the study, for example offering consent and baseline assessments at home visits instead of sending them by post to reduce queries and improve data completeness. In future studies, we will pre-specify progression criteria, standardise a minimum dose, publish materials and core components via TIDieR, and use a fidelity checklist.

Pragmatic hybrid delivery, home plus video, appeared workable for families and clinicians, matching evidence that telehealth can maintain fidelity and reduce barriers to access in older cohorts. Harms monitoring recorded no serious adverse events. The CONSORT Harms recommendations call for explicit reporting of all unintended effects, even minor ones (such as needing to reschedule school attendance for home sessions). We will incorporate this fully into the reporting procedures for the full trial.

Publishing ACHIEVE’s core components, tailoring rules, and fidelity approach will make replication and commissioning easier. Embedding an implementation lens will yield results that are not only clinically credible but also actionable for commissioners and service leaders weighing scale-up across diverse NHS and education settings.

Our feasibility trial has a number of limitations. First, this was a single-region feasibility study without a control group or randomisation, reliant on parent-report outcomes and with limited scope for blinded assessment given the home-based delivery. This means effectiveness cannot be inferred and observed changes may reflect non-specific effects or natural variation. Second, although fidelity was supported through documentation and supervision, we did not undertake independent fidelity assessment, limiting certainty about consistency of delivery. Third, recruitment through participating schools constituted convenience sampling and may limit generalisability beyond similar settings. Finally, we did not quantify baseline concomitant interventions or parent expectations in a way that allows adjustment, although this is an underserved population with limited access to interventions.

School-mediated identification was efficient and resulted in a varied and representative sample but may have narrowed reach. Future studies could widen recruitment through agencies such as voluntary sector partners. Our choice of measures proved burdensome for some families resulting in some incomplete data, which has impacted our ability to assess progress. Future studies that incorporate families with very variable language backgrounds, SES and literacy should anticipate providing translation and interpreting services, and more direct support for families, with financial incentives for data completion. None of these constraints undermines the aim of the phase: to test delivery, engagement, and measurement and to learn what to fix or standardise before a definitive, comparative evaluation.

ACHIEVE sits within current debates about how health, education, and social care services can provide earlier, practical support to families of adolescents with severe learning disabilities. Families often provide most long-term care, and unmet need during adolescence can affect wellbeing, participation, and transition to adulthood. Previous research has shown the impact on families when community support is insufficient and needs escalate to residential provision [7], and transition studies highlight the complexity experienced by families as young people with intellectual disability move toward adult services [34]. The evidence base for parent-focussed interventions in adolescents with intellectual disabilities is promising but limited: a recent systematic review reported benefits for parenting, parent–adolescent relationships, and wellbeing but also highlighted mixed study quality and little cost-effectiveness evidence [21]. ACHIEVE therefore contributes feasibility, acceptability, dose, and implementation evidence for a home-based, parent-coached OT/SLT model. These findings should inform a future controlled trial with process and economic evaluation rather than be interpreted as evidence of effectiveness.

Building on ACHIEVE’s feasibility signals, future studies should consider two-arm, multi-school randomised controlled trials with a wait-list control. A controlled design is important to estimate effectiveness and to mitigate biases such as secular change and expectancy effects. Therapists and families would be unblinded to allocation, but outcome assessors should remain blinded, with a detailed pre-published protocol specifying intention-to-treat analyses. Our data suggest that a nominated primary outcome could measure parental empowerment, control, and confidence within their family and local community, e.g. the Family Empowerment Scale. Other measures could be retained such as the child-focussed measures (DBC) and parent outcomes (Parental Stress Scale, HSQ). The therapist completed measures: COPM and goal attainment scaling were valuable in tracking goal-level change. Study designs could consider wait-list control, pragmatic cluster, or stepped-wedge design while preserving blinded assessment and consistent outcome timing.

To conclude, a home-based, parent-coached programme for adolescents with learning disabilities was feasible in UK practice. We found high attendance, strong alliance, and acceptable data capture, with exploratory improvements that help set dose and measurement for a larger study. The next step is a definitive, comparative evaluation that proceeds with clear progression criteria, fidelity checks, broader and more equitable recruitment, transparent harm reporting, and an explicit implementation framework for scale-up.

## Data Availability

De‑identified data and analytic code are available on reasonable request—consistent with institutional and funder policies.

## Abbreviations

LD: Learning disabilities
ASD: Autism spectrum disorder
OT: Occupational therapy
SLT: Speech and language therapy
WAI: Working alliance inventory
DBC: Developmental behaviour checklist
HSQ: Home situations questionnaire
APSI: Autism parenting stress index
COPM: Canadian occupational performance measure
GAS: Goal attainment scaling
TIDieR: Template for intervention description and replication
CONSORT: Consolidated standards of reporting trials
IMD: Indices of multiple deprivation
